# Dr. E. L. Detwiler

**Published:** 1916-05

**Authors:** 


**Dr. E. L. Detwiler** of Herndon, Va., was shot dead by the son of a patient on Feb. 29, for advising sending the patient to a hospital. The slayer is charitably supposed to have been insane. This is a strange illustration of the manifold dangers of the medical profession; yet not so strange after all, when we consider the strong opposition and manifestation of sentiment often encountered when such advice is given. One of our late colleagues was attacked by the husband of a woman who died in childbirth, and we have known of several instances of attacks by dogs during professional calls. Frequently we read of murders of physicians on account of jealousy, sometimes apparently justified, usually without reasonable grounds. We all realize the dangers of medical practice from exposure and fatigue and from accidents during transit to and from patients. Railroad and automobile accidents are of almost weekly occurrence; runaways, drowning in streams that have carried away bridges, freezing, etc., are now more common in fiction than real life. It is worth while to consider that the medical profession runs an appreciable risk from manslaughter, directly due to professional occupation.

